# Pathogenicity of Autoantibodies in Anti-p200 Pemphigoid

**DOI:** 10.1371/journal.pone.0041769

**Published:** 2012-07-24

**Authors:** Katerina Vafia, Stephanie Groth, Tina Beckmann, Misa Hirose, Jenny Dworschak, Andreas Recke, Ralf J. Ludwig, Takashi Hashimoto, Detlef Zillikens, Enno Schmidt

**Affiliations:** 1 Department of Dermatology, University of Luebeck, Luebeck, Germany; 2 Department of Dermatology, Kurume University School of Medicine, Kurume, Japan; 3 Comprehensive Center for Inflammation Medicine, University of Luebeck, Luebeck, Germany; University of Colorado Denver, United States of America

## Abstract

Recently, the C-terminus of laminin γ1 has been identified as target antigen in anti-p200 pemphigoid and the disease was renamed as anti-laminin γ1 pemphigoid. However, the pathogenic relevance of these autoantibodies has not yet been demonstrated. Therefore, we employed an ex vivo model of autoantibody-mediated leukocyte-dependent neutrophil activation and dermal-epidermal separation (DES) using cryosections of human skin. We showed that anti-p200 pemphigoid sera (n = 7) induced DES in a time-dependent manner, in contrast to sera from healthy controls. Furthermore, laminin γ1-specific IgG and serum depleted from anti-laminin γ1 reactivity were generated using the recombinant C-terminus of laminin γ1 (LAMC1-term; amino acids 1364 to 1609). Interestingly, both fractions labeled the dermal-epidermal-junction (DEJ) by indirect immunofluorescence microscopy on human foreskin and recognized a 200 kDa protein by immunoblotting with dermal extract. Human and rabbit IgG against LAMC1-cterm failed to attract neutrophils at the DEJ and to induce DES. In contrast, patient serum depleted from LAMC1-cterm reactivity led to the same extent of DES as non-depleted IgG. Repeated injection of rabbit anti-murine LAMC1-cterm IgG into both neonatal and adult C57BL/6mice as well as repetitive immunization of various mouse strains with murine LAMC1-cterm failed to induce macro- and microscopic lesions. In all mice, circulating anti-LAMC1-cterm antibodies were present, but only in some mice, IgG deposits were seen at the DEJ. We conclude that autoantibodies in anti-p200 pemphigoid sera are pathogenic while pathogenicity is not mediated by autoantibodies against laminin γ1. Further studies are needed to identify the pathogenically relevant autoantigen in anti-p200 pemphigoid.

## Introduction

Anti-p200 pemphigoid is an autoimmune subepidermal blistering disease which was first described in 1996 [1], [2]. Clinically, the disease is characterized by tense blisters and resembles bullous pemphigoid, the most frequent autoimmune blistering disease although patients with anti-p200 pemphigoid tend to be younger [3]. Autoantibodies in patients’ skin localize along the dermal-epidermal junction (DEJ) by direct immunofluorescence (IF) microscopy. Serum IgG autoantibodies label the dermal side of 1 M NaCl-split human skin by indirect IF microscopy and recognize a 200 kDa protein by immunoblotting of human dermal extract [1], [2]. Subsequently, the target antigen was characterized as an acidic non-collagenous N-linked glycoprotein of the lower lamina lucida [1], [2], [4], [5]. Recently, Dainichi *et al.* showed reactivity with anti-laminin γ1 in about 90% of patients’ sera and coined the term anti-laminin γ1 pemphigoid [6], [7]. Furthermore, the C-terminus of laminin γ1 was identified as the immunodominant region of this protein, a finding that we recently confirmed by developing an ELISA using a recombinant monomeric C-terminal fragment of laminin γ1 [8].

So far, nothing is known about the pathogenic relevance of anti-laminin γ1 autoantibodies. We and others have previously developed various experimental models that demonstrated the pathogenic relevance of autoantibodies in different subepidermal blistering autoimmune disorders using passively transferred IgG [9]–[15]. More specifically, we previously developed an ex vivo model in which incubation of IgG from patients with bullous pemphigoid (BP) and epidermolysis bullosa acquisita (EBA) or rabbit antibodies raised against the target antigens BP180 (type XVII collagen) and type VII collagen, respectively, induced leukocyte-dependent dermal-epidermal separation in cryosections of human skin [10], [16]. Furthermore, both the injection of anti-murine type VII collagen and the immunization with recombinant murine type VII and type XVII collagen led to blistering phenotypes in adult mice closely mimicking EBA and BP, respectively [11], [17], [18].

In the present study, serum from patients with anti-p200 pemphigoid induced dermal-epidermal splitting in cryosections of human skin. In contrast, patient IgG affinity-purified against the recombinant C-terminus of human laminin γ1 (hLAMC1-cterm), and a C-terminal fragment of laminin 111 [6] and the whole laminin γ1 chain, respectively, failed to induce dermal-epidermal separation in this model. In addition, total IgG and IgG affinity-purified using recombinant mLAMC1-cterm generated from mLAMC1-cterm-immunized rabbits, respectively, were ineffective in the ex vivo cryosection model and did not cause macro- and microscopic disease after injection into both neonatal and adult mice. Furthermore, immunization of mice with mLAMC1-cterm induced mLAMC1-cterm-specific autoantibodies but did not result in clinical disease. These studies indicate that autoantibodies in anti-p200 pemphigoid are pathogenic ex vivo but autoantibodies against the C-terminus of laminin γ1 do not mediated pathogenicity in this disease.

## Materials and Methods

### Human Sera and Anti-laminin γ1 Antibodies

Serum samples were obtained from patients with anti-p200 pemphigoid (n = 25) and characterized as described before [8]. The study was approved by the ethics committee of the University of Luebeck (11-143). Written informed consent was obtained from all patients seen in our department. The majority of sera were obtained anonymously from the routine autoimmune laboratory of our department to which they were sent from various hospitals worldwide. The anonymous use of patients' sera left over after routine diagnosis has been approved by our local ethics committee for patients that were not seen in our department (11-143). Rabbits SA6539 and SA6794 were generated against recombinant murine (m)LAMC1-cterm. IgG affinity-purified from a BP patient serum with anti-BP180 NC16A reactivity, rabbit IgG against murine BP180 NC15A, and preimmune rabbit serum were used as controls. Polyclonal rabbit anti-hLAMC1 crossreacting with mLAMC1 (H-190) and mouse monoclonal anti-hLAMC1 (clone B-4) were purchased from Santa Cruz Biotechnology (Santa Cruz, CA, USA).

### Mice

C57BL/6, BALB/c, and SJL mice were obtained from Charles River Laboratories (Sulzfeld, Germany). All injections and bleedings were performed on mice anesthetized by intraperitoneal administration of a mixture of ketamine (100 µg/g) and xylazine (15 µg/g). Experiments were approved by the Animal Rights Commission of the Ministry of Agriculture and Environment, Schleswig-Holstein (V312-72241.122-5(79-6/09) and V312-72241.122-5(80-6/09)).

### Preparation of Dermal Extracts

Murine dermal extracts were prepared as described for human skin [1] The dermal extract was then stored in −80°C.

### Cloning, Expression and Purification of Recombinant Fragments of LAMC1

**Figure 1:**
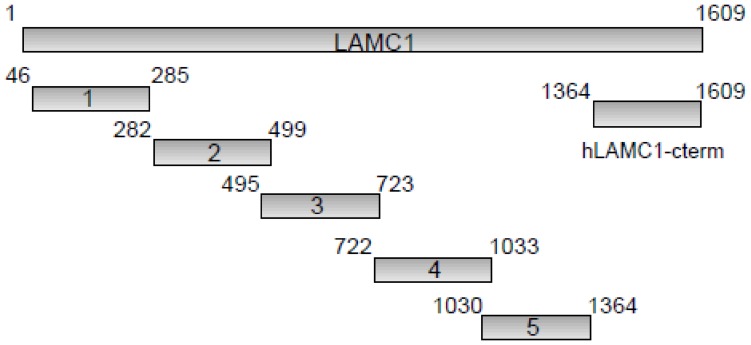
Schematic diagram of the 6 recombinant fragments of human laminin γ1 (hLAMC1) used in this study. The recombinant C-terminus (hLAMC1-cterm) was previously used as antigenic target in an ELISA for the diagnosis of anti-p200 pemphigoid [8]. Each recombinant fragment is fused with an N-terminal His-tag. Amino acid numbers are shown next to the fragments.

Human LAMC1-cterm (hLAMC1-cterm; amino acids 1364 to 1609) was expressed in *E.coli* as described previously [8] and in the human cell line HEK293T (Cell Lines Service, Eppelheim, Germany). For latter expression, the sequence included a signal peptide from Ig kappa for secretion into the culture medium. HEK293T cells cultured in DMEM (Invitrogen, Karlsruhe, Germany) with 10% fetal calf serum were transfected with hLAMC1-cterm-pEE14.4 (Lonza, Cologne, Germany) After 48 h, hLAMC1-cterm was purified by immobilized metal affinity chromatography on TALON superflow (Clontech, Palo Alto, CA, USA). Furthermore, *5* overlapping fragments of hLAMC1 comprising the portion not covered by hLAMC1-cterm were expressed as His-fusion proteins in the *E.coli* strain Rosetta DE3 (Fig. 1). DNA sequence data for human LAMC1 was retrieved from GenBank (accession number: NM-002293). Primer pairs used in standard PCR reaction are shown in table 1 (VBC-Biotech, Vienna, Austria). The amplification products were subcloned into pQE40, using a BamH*I/*Hind*III* cutting site. Expression and purification were performed as described above as described previously [8]. Previous study showed that anti-p200 pemphigoid patients’ sera are not reactive with murine skin [19]. Therefore we generated the C-terminal fragment (mRNA: 4330-5069 bp, 740 bp, accession number NM- 010683) of murine laminin γ1 (mLAMC1-cterm) which was optimised for expression in *E*. *coli* (Mr. Gene, Regensburg, Germany). Murine LAMC1-cterm was subcloned from the obtained vector mLAMC1-cterm-pMA into the expression vector pQE-40 as described for hLAMC1-cterm [8].

**Table 1:** Primer sequences for PCR amplification of cDNA fragments of human.

	Size (bp)	Primer sequence (5‘-3‘)
1	740	F: ATGGATCCCGGCCGCAGCGCTGCA
		R:AGCGGTACCTCATCTGCCACCTACAGCAAAATCAGAGA
2	670	F: ATGGATCCACTGTAGGTGGCAGATGTAAATGTAATG
		R: ATGGTACCTCAGCAGAAGCAGGGTGTGCAACC
3	706	F: ATGGATCCTGCTTCTGCTTTGGGCATTCT
		R: ATGGTACCTCAAAGCACACATGGACTGTATGGTC
4	958	F: ATGGATCCAGTGTGCTTTGCGCCTGCAATG
		R: ATGGTACCTCAGGCTGGACATTCCTGGCAGCCAG
5	1021	F:ATGGATCCGAATGTCCAGCTTGTTACCGG
		R: ATGGTACCTCAAGCTTCTTGTAAGGTATCCCG

F, forward primer; R, reverse primer.

The recombinant E8 fragment of laminin 111, a heterotrimer of the truncated C-terminal portions of α1, β1, and γ1 chains was kindly provided by Dr. Sekiguchi (Institute for Protein Research Osaka University) [6]. The plasmid pTriEx-1 containing full length human laminin γ1 (hLAMC1-FL) was kindly provided by Euroimmun AG. Transfection of HEK293T cells and purification of hLAMC1-FL was performed as for hLAMC1-cterm described above.

### Immunoblotting

Recombinant proteins and dermal extracts were fractionated by SDS-PAGE, transferred to nitrocellulose membrane and immunoblotted as reported [8]. Human (1∶50), mouse (1∶50), and rabbit sera (1∶1,000), polyclonal rabbit anti-hLAMC1 crossreacting with mLAMC1 (clone H-190) and mouse monoclonal anti-hLAMC1 (clone B-4; both 1∶200) were diluted in TBST containing 5% skimmed milk powder plus 1% BSA. As secondary antibody horseradish peroxidase (HRP)-conjugated monoclonal mouse anti-human IgG4 antibody (Southern Biotech, Birmingham, Alabama, USA), polyclonal rabbit anti-mouse IgG antibody (DAKO, Hamburg, Germany) and polyclonal goat anti-rabbit antibody (DAKO) were used.

### Affinity Purification of Rabbit and Human IgG

IgG from rabbit sera was isolated using Protein G Sepharose Fast Flow affinity column chromatography (GE Healthcare, Munich, Germany) as described previously [20]. Concentrations of IgG were determined using BCA assay (Thermo Scientific, Rockford, USA). Antibodies to hLAMC1-cterm (prokaryotic and eukaryotic expressed forms), E8 fragment of laminin 111, as well as antibodies to hLAMC1-FL and mLAMC1-cterm were affinity-purified from sera of anti-p200 pemphigoid patients and rabbits immunized with mLAMC1-cterm, respectively, using Affi-Gel 15 (Bio-Rad, Munich, Germany) and the MicroLink™ Protein Coupling Kit (Thermo Fisher Scientific p/a Pierce Biotechnology, Rockford, USA) following the manufacturer’s instructions. Autoantibodies to hLAMC1-FL were generated using the sera affinity-purified against eukaryotic expressed hLAMC1-cterm protein followed by incubation with the immobilized hLAMC1-FL protein. After both steps of affinity-purification, eluted antibodies were pooled and used for the experiments.

### Cryosection Assay

Blister-inducing capacity of patients’ autoantibodies and rabbit IgG was evaluated using the cryosection assay, an *ex vivo* model of autoantibody-induced dermal-epidermal separation originally described by Gammon *et al.* [21] and modified by Sitaru *et al.* [10], [16]. Briefly, cryosections of neonatal human foreskin and mouse tail were incubated for one hour at 37°C with 50 µl of patients’ and rabbit sera (dilution 1∶3 in PBS). In addition, hLAMC1-cterm-specific IgG and sera depleted from anti-hLAMC1-cterm reactivity were used after adjusting to serum IgG concentrations. Then, leukocyte suspension from healthy volunteers isolated by dextran 500 (ROTH, Karlsruhe, Germany) sedimentation mixed with medium (RPMI, LONZA, Cologne, Germany) was incubated with the skin sections for 3 hours at 37°C. After washing with PBS slides were fixed in formalin and stained with haematoxylin and eosin. Sections were examined by two blinded independent investigators at x200 magnification. Analogous experiments were performed using the E8 fragment of laminin111 and hLAMC1-FL.

### Mouse Experiments

In the passive transfer model, purified rabbit IgG generated against mLAMC1 and preimmune rabbit IgG (15 mg per adult mouse and 10 mg/g per neonatal mouse) as well as affinity-purified rabbit antibodies specific to mLAMC1-cterm (1 mg/g per neonatal mouse) were injected subcutaneously in abdominal skin every second day for 12 days. On day 14, mice were sacrificed, and samples (ears, tails, blood) were taken for further analysis. In the immunization-induced model, mice were immunized subcutaneously into footpads 4 times (with 3 weeks interval) with 60 µg of purified mLAMC1-cterm emulsified in adjuvant (TiterMax®, Alexis, Lörrach, Germany). Mice were examined every second week for evidence of cutaneous lesions (i.e., erythema, blisters, erosions, or crusts). Control mice were immunized with PBS and TiterMax®. All mice were observed for at least 16 weeks. From every mouse, serum and tissues samples were obtained at weeks 2, 4, 6, 8, and 16 for further analysis.

### Immunofluorescence Microscopy

For direct IF microscopy, 6 µm sections of mouse skin were incubated with FITC-labelled polyclonal rabbit anti-mouse IgG (1∶100, DAKO) and anti-murine C3 IgG (1∶50, Cappel Organon-Teknika, Durham, NC). For indirect IF microscopy, 6 µm sections of human and mouse skin were incubated with human, rabbit, and mouse sera (diluted 1∶50 in PBS) and for detection, FITC-labelled monoclonal anti-human IgG4 (1∶50, Sigma Aldrich, Munich, Germany) and polyclonal swine anti-rabbit IgG (1∶100, DAKO) were employed.

### Anti-mLAMC1-cterm ELISA

Each well of 96-well microtiter plates (MaxiSorp, Nunc, Roskilde, Denmark) was coated with 4 µg/ml mLAMC1-cterm in PBS at 4°C over night. After blocking with PBST containing 5% skimmed milk, wells were incubated with a 100-fold dilution of mouse sera for 1 h. Bound antibodies were detected using an HRP-conjugated polyclonal rabbit anti-mouse IgG antibody (DAKO) and polyclonal goat anti-rabbit antibody (DAKO) diluted 1∶2,000 in blocking buffer followed by addition of 1-Step Turbo TMB-ELISA solution (Fisher Scientific, Schwerte, Germany) for 1–3 minutes. The OD_450nm_ was measured using a VICTOR3 Wallac 1420 microplate reader (Perkin-Elmer LAS, Rodgau, Germany). All steps were carried out at room temperature. All sera were tested in duplicates. From the mean OD value for each serum sample, the mean OD value of the blank (PBST) was subtracted.

## Results

### Sera from anti-p200 Pemphigoid Patients Induce Subepidermal Splitting in Cryosections of Human Skin

**Figure 2:**
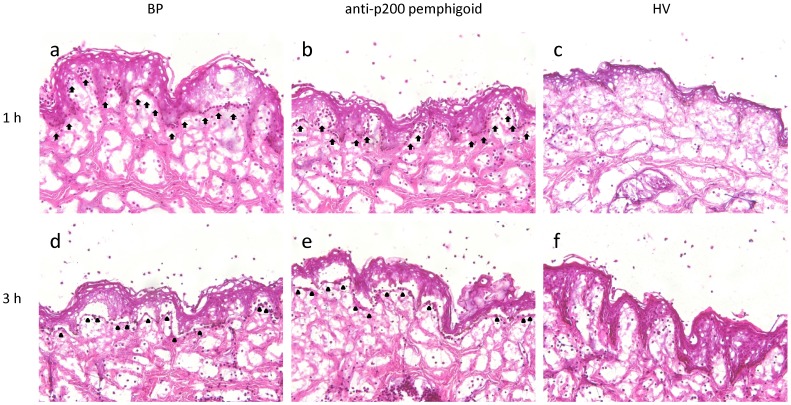
Anti-p200 pemphigoid patients’ sera induce dermal-epidermal separation ex vivo. Sera from anti-p200 pemphigoid patients recruit neutrophils to the dermal-epidermal junction (DEJ) and induce dermal-epidermal separation (DES) in cryosections of human skin. Anti-p200 pemphigoid sera (anti-p200 pemphigoid; b, e), but not from sera of healthy volunteers (HV; c, f), recruited leukocytes to the DEJ after 1 h of incubation (b, c) and induced DES after 3 h (e, f). As positive control for DES, IgG from a patient with bullous pemphigoid was used (BP a, d). Recruited neutrophils are marked by arrows, base of the split is marked by black triangles. All sections were stained with hematoxylin and eosin. Magnification, x200.

Serum samples from anti-p200 pemphigoid patients (n = 7) as well as sera from a patient with BP and from healthy volunteers were incubated with cryosections of human skin in the presence of leukocytes purified from healthy donors. Autoantibodies from all tested patients bound to the DEJ and led to recruitment of leukocytes (Fig. 2a, b), followed by dermal-epidermal separation (DES) after a 3 h incubation with leukocytes (Fig. 2d, e). Sera from healthy volunteers served as controls (Fig. 2c, f).

### Anti-hLAMC1-specific IgG from Patients with anti-p200 Pemphigoid does not Induce Split Formation in Cryosections of Human Skin

**Figure 3:**
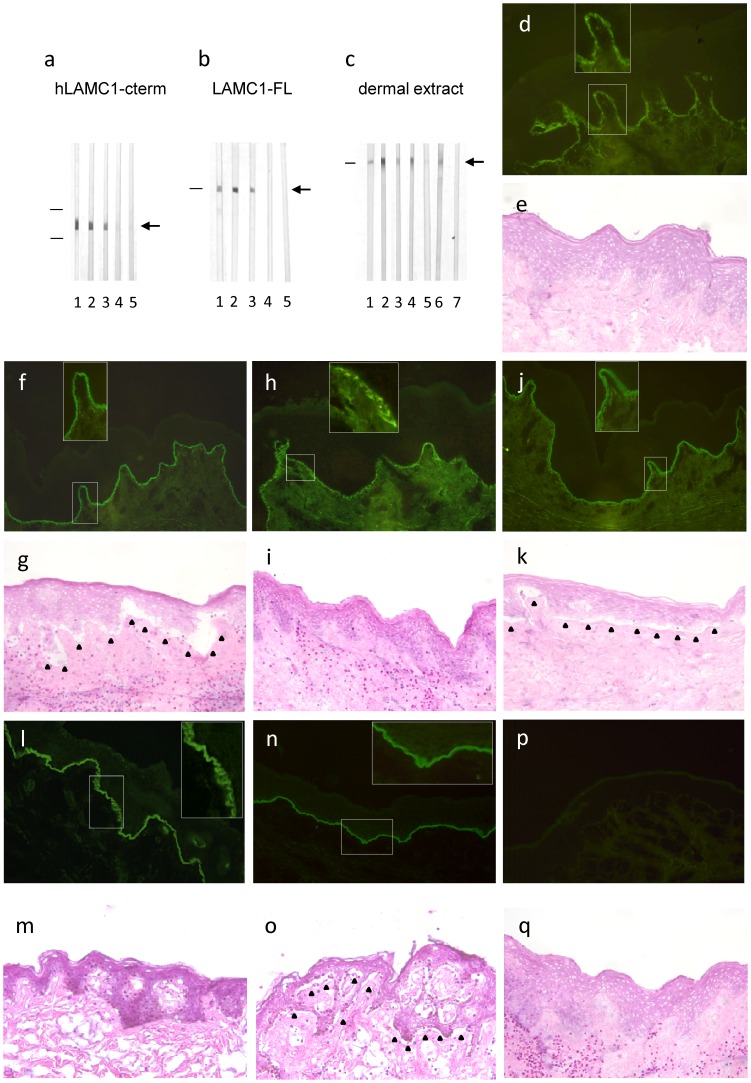
Human IgG specific to laminin γ1 is not pathogenic ex vivo. Using recombinant forms of the C-terminus of laminin γ1 (hLAMC1-cterm) and full length laminin γ1 (LAMC1-FL) IgG specific for hLAMC1-cterm (a, c; lane 3) and LAMC1-FL (b, lane 3; c lane 5 ) was generated from anti-p200 pemphigoid serum (a, b, c; lane 2), as well as serum depleted from anti-hLAMC1-cterm (a, c, lane 4) and LAMC1-FL reactivity (b, lane 4; c lane 6) reactivity, respectively, as shown by immunoblotting with recombinant hLAMC1-cterm (a), LAMC1-FL (b), and extract of human dermis-(c). Interestingly, serum depleted from anti-hLAMC1-cterm and LAMC1-FL reactivity, respectively, (a, b; lane 4) still labeled the p200 protein in dermal extract (c, lane 4, lane 6). Monoclonal antibody against LAMC1 (a, b, c; lane 1) and serum from a healthy volunteer (a, b; lane 5; c lane 7) were used as controls. Arrows indicate the positions of the proteins and bars the molecular weight markers (a, 34 kD and 26 kD; b, 200 kD; c, 200 kD). hLAMC1-cterm-specific (h, i) and LAMC1-FL-specific patients IgG (l, m) and the monoclonal anti-LAMC1 antibody (d, e) labeled the dermal-epidermal junction (DEJ) by indirect immunofluorescence (IF) microscopy but did not induce dermal-epidermal separation (DES). In contrast, serum depleted from reactivity against hLAMC1-cterm (j, k), and LAMC1-FL (n, o), respectively, as well as patient serum (f, g) resulted in DES (black triangles mark base of the split). While untouched patient serum (f) as well as serum depleted from anti-hLAMC1-cterm (j) and LAMC1-FL reactivity (n), respectively, stained the DEJ of human skin in a linear pattern, the monoclonal anti-hLAMC1-antibody (d), hLAMC1-cterm-specific patient-IgG (h), and hLAMC1-FL-specific patient IgG showed an additional staining of basal keratinocytes. Serum from a healthy volunteer was used as control (p, q). Magnification: x200.

To explore the pathogenic effect of anti-laminin γ1 antibodies, IgG from anti-p200 pemphigoid patients was affinity-purified using four different recombinant antigens: (i) hLAMC1-cterm expressed in *E.coli*, (ii) hLAMC1-cterm expressed in HEK293 cells, (iii) E8 fragment covering the C-terminus of laminin 111, and (iv) the entire hLAMC1-FL molecule. hLAMC1-cterm-specific IgG of all 5 tested sera as well as the monoclonal anti-LAMC1 antibody (B-4) recognized both recombinant hLAMC1-cterm and the p200 antigen by immunoblotting (Fig. 3a, c) and labelled the DEJ by indirect IF microscopy on human skin (Fig. 3h, d), but did not induce splitting in cryosections of human skin (Fig. 3i, e). In contrast, all 5 sera depleted from anti-hLAMC1-cterm reactivity labelled the p200 antigen by immunoblotting and the DEJ by indirect IF microscopy of human skin, respectively (Fig. 3a, c, j), but induced DES in the cryosection model (Fig. 3k). While in addition, anti-p200 pemphigoid serum depleted of total IgG did not induce DES in the cryosection model (data not shown). To exclude suboptimal dosing, hLAMC1-cterm-specific IgG was applied at a 5-fold higher concentration compared to the serum. The concentrated hLAMC1-cterm-specific IgG did also not induce DES.

Since laminin γ1 is known to be N-glycosylated [5], in the next set of experiments, IgG from anti-p200 pemphigoid patients (n = 3) was affinity-purified using recombinant hLAMC1-cterm expressed in HEK293 cells. The same set of experiments was also performed using the recombinant E8 fragment (n = 3). In both cases the obtained hLAMC1-cterm-specific IgG reacted with the p200 protein in dermal extract but did not induce DES in the cryosection model. In contrast, incubation of serum depleted from reactivity with eukaryotic expressed hLAMC1-cterm still resulted in DES (data not shown). In a final approach, the entire protein hLAMC1-FL was employed in 3 patient sera. Again, the affinity-purified IgG reacted with the p200 protein in dermal extract (Fig. 3c) but did not induce DES (Fig. 3m) while serum depleted from hLAMC1-FL reactivity induced splitting (Fig. 3o).

By indirect IF microscopy on human skin, in addition to a linear binding pattern, staining of the basal keratinocytes was noticed with (i) the monoclonal anti-LAMC1 antibody (Fig. 3d, insert), (ii) patient hLAMC1-cterm-specific IgG (Fig. 3h, insert), and (iii) patient hLAMC1-FL-specific IgG (Fig. 3l). In contrast, patient sera and sera depleted from anti-hLAMC1-cterm and hLAMC1-FL antibodies, respectively, revealed a linear staining throughout the specimen (Fig. 3f, h, n; inserts).

We concluded that autoantibodies to laminin γ1 did not mediate DES induced by anti-p200 pemphigoid sera in the cryosection model.

### Epitope Mapping of the Entire hLAMC1

To test the hypothesis that major antigenic sites are present on laminin γ1 outside hLAMC1-cterm, 5 overlapping recombinant fragments covering the entire laminin γ1 molecule outside hLAMC1-cterm were expressed in *E. coli* (Fig. 1). Twenty-five anti-200 pemphigoid sera, including 21 sera that recognized hLAMC1-cterm, were probed for reactivity with the 5 laminin γ1 fragments by immunoblotting. Only weak reactivity outside hLAMC1-cterm was detected in 32% of patients’ sera. Highest reactivity was found with fragment 5 in 4 (16%) of 25 sera (Table 2).

**Table 2:** Serum autoantibody reactivity in anti-p200 pemphigoid patients with overlapping fragments of laminin γ1 covering the whole molecule.

	fragments outside hLAMC1-cterm					hLAMC1-cterm
	1	2	3	4	5	
positive sera	1 of 25	2 of 25	3 of 25	1 of 25	4 of 25	21 of 25
	8 of 25					

### Pathogenicity of Rabbit IgG Against Recombinant Murine LAMC1-cterm (mLAMC1-cterm)

**Figure 4:**
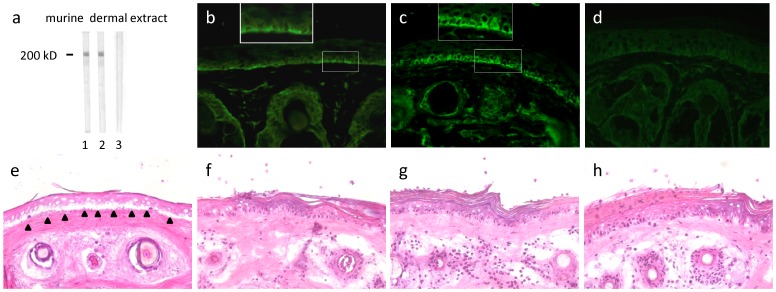
A high concentration of rabbit anti-mLAMC1-cterm IgG is not pathogenic ex vivo. Rabbit IgG generated against the murine laminin γ1 C-terminus (mLAMC1-cterm) did not induce DES in cryosections of mouse skin. Rabbit anti-mLAMC1-cterm (a, lane 2; c, g; dilution 1∶1000) and commercial rabbit antibody H-190 against mLAMC1 (a, lane 1; b, f, dilution 1∶200) recognized the p200 protein by immunoblotting with extract of murine dermis (a) and labeled the DEJ of murine skin (b, c) but did not induce DES in cryosections of mouse skin (f, g). Of note, anti-mLAMC1-cterm rabbit IgG stained the basal layer of keratinocytes at the murine DEJ (b, c, inserts) in a similar pattern as seen with monoclonal anti-hLAMC1-antibody and hLAMC1-cterm-specific patient IgG (Fig. 3, c-f). Rabbit IgG against murine BP180 NC15A (e) and preimmune rabbit IgG (d, h) were used as positive and negative controls, respectively. Magnification: x400.

Rabbit anti-mLAMC1-cterm IgG reacted with a 200 kDa protein in the extract of murine dermis by immunoblotting (Fig. 4a lane 2). The same band was labelled by the commercial polyclonal anti-murine LAMC1 antibody H190 (Fig. 4a lane 1) that also recognized the recombinant mLAMC1-cterm used for immunization of rabbits (data not shown). Furthermore, rabbit anti-mLAMC1-cterm IgG stained the DEJ of mouse skin at a titre of 1∶100 by indirect IF microscopy. The staining of the basal keratinocytes (Fig. 4c) was similar to the pattern seen with patient hLAMC1-cterm specific IgG on human skin (Fig. 3e, insert). Faint binding was also observed with the polyclonal anti-LAMC1 antibody H190 by indirect IF microscopy on murine skin (Fig. 4b). When used in the cryosection model, rabbit anti-mLAMC1-cterm IgG did not recruit neutrophils at the DEJ and did not induce DES even when applied at very high titres of >1∶10^5^ by immunoblotting with dermal extract. (Fig. 4g). Same findings were obtained using the monoclonal LAMC1 antibody H190 (Fig. 4f) and preimmune rabbit IgG (Fig. 4h). In contrast, as shown previously [10], rabbit anti-mBP180 NC15A IgG (reactive against the immunodominant region of BP antigen) resulted in DES (Fig. 4e).

#### Passive transfer of IgG to neonatal mice

**Figure 5:**
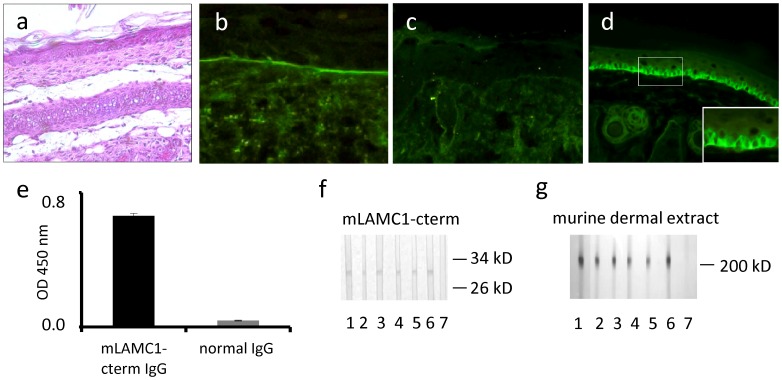
Passive transfer of rabbit anti-mLAMC1-cterm IgG into neonatal mice does not reproduce the human disease. Rabbit IgG against the murine laminin γ1 C-terminus (mLAMC1-cterm) was not pathogenic when passively transferred into neonatal C57BL/6 mice. Injection of rabbit anti-mLAMC1-cterm IgG at a concentration of 10 mg/g body weight every second day for 10 days did not induce histopathological lesions on day 12 (a). Linear deposition of rabbit IgG at the DEJ was only observed in 2 of 8 mice (b), while staining of murine C3 was always negative (c). At day 12, in sera of all mice, rabbit IgG stained the basal keratinocytes at the DEJ of normal mouse skin (d), reacted with recombinant mLAMC1-cterm by ELISA (e) and immunoblotting (f, lanes 2–6) and with the 200 kDa p200 protein in extract of murine dermis (g, lanes 2–6). Polyclonal rabbit antibody H-190 against mLAMC1 (f, g, lane 1) and normal mouse serum (f, g, lane 7) was used as controls.

To explore the pathogenic effect of IgG from rabbits immunized against mLAMC1-cterm *in vivo,* neonatal C57BL/6 mice (n = 8) were injected with rabbit anti-mLAMC1-cterm IgG and preimmune rabbit IgG, respectively, at a concentration of 10 mg/g body weight. In none of the mice, clinical disease was observed and histopathological examinations of back skin did not reveal dermal inflammation or subepidermal blisters (Fig. 5a). Direct IF microscopy of back skin showed linear deposits of rabbit IgG in 2 out of 8 mice, while in none of the mice, deposits of murine complement C3 were detected at the DEJ (Fig. 5b, c). All sera taken on day 12 showed IgG staining of basal keratinocytes along the DEJ by indirect IF microscopy of normal mouse skin at titres between 1∶30 and 1∶40 (Fig. 5d). In all mice, strong anti-mLAMC1-cterm reactivity was also detected by ELISA (Fig. 5e) and immunblotting with recombinant mLAMC1-cterm and the 200 kDa protein in extract of murine dermis (Fig. 5f, g). Also anti-mLAMC1-cterm specific IgG affinity-purified with recombinant mLAMC1-cterm from rabbits immunized with mLAMC1-cterm were injected into neonatal mice (n = 3) at a concentration of 1 mg/g of body weight with similar outcome (data not shown).

#### Passive transfer of IgG into adult mice

**Figure 6:**
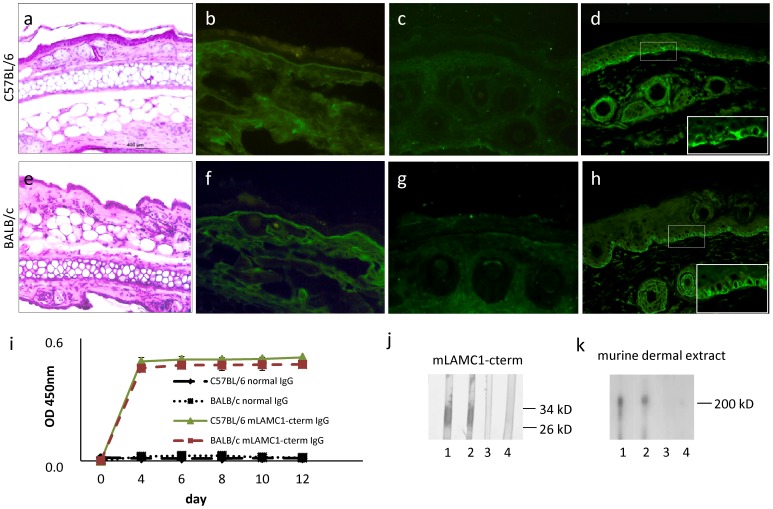
Passive transfer of rabbit anti-mLAMC1-cterm IgG into adult mice is not pathogenic. Rabbit IgG against the murine laminin γ1 C-terminus (mLAMC1-cterm) was not pathogenic when passively transferred into adult C57BL/6 and BALB/c mice. Injection of 15 mg rabbit anti-mLAMC1-cterm IgG every second day for 10 days did not result in clinical or histopathological (a, e) lesions on day 12. Linear deposition of rabbit IgG at the dermal-epidermal junction (DEJ) was only observed in 2 of 5 C57BL/6 (b) and one of 5 BALB/c mice (f), while staining of murine C3 was negative in all mice (c, g). At day 12, in sera of all 10 mice, rabbit IgG labeled the basal keratinocytes at the DEJ of normal mouse skin (d, h) and reacted with recombinant mLAMC1-cterm by ELISA (i) and immunoblotting (j, C57BL/6, lanes 1; BALB/c, lane 2) and the 200 kDa p200 protein in extract of murine dermis (k, C57BL/6 lane 1; BALB/c, lane 2). Normal mouse sera (j and k, C57BL/6, lane 3; BALB/c, lane 4) were used as controls.

C57BL/6 (n = 5) and BALB/c (n = 5) adult mice were injected, every second day, for 12 days, with 15 mg of rabbit anti-mLAMC1-cterm IgG. None of the mice treated showed blisters clinically or histopathologically (Fig. 6a, e). IF microscopy of perilesional mouse skin revealed weak, linear, deposits of rabbit IgG in 2 C57BL/6 and one BALB/c mice (Fig. 6b, f), while murine complement C3 at the DEJ was negative in all cases (Fig. 6c, g). All mice showed serum autoantibodies against the DEJ with titers ranging from 1∶30 to 1.40 by indirect IF microscopy (Fig. 6d, h), to p200 by immunoblotting (titre 1∶10^5^) (Fig. 6k), and to mLAMC1-cterm (titre 1∶10^5^) by ELISA and immunoblotting (Fig. 6 i, j).

### Immunization Against mLAMC1-cterm Induces Autoantibody Production in mice of Different Strains but no Blisters

**Figure 7:**
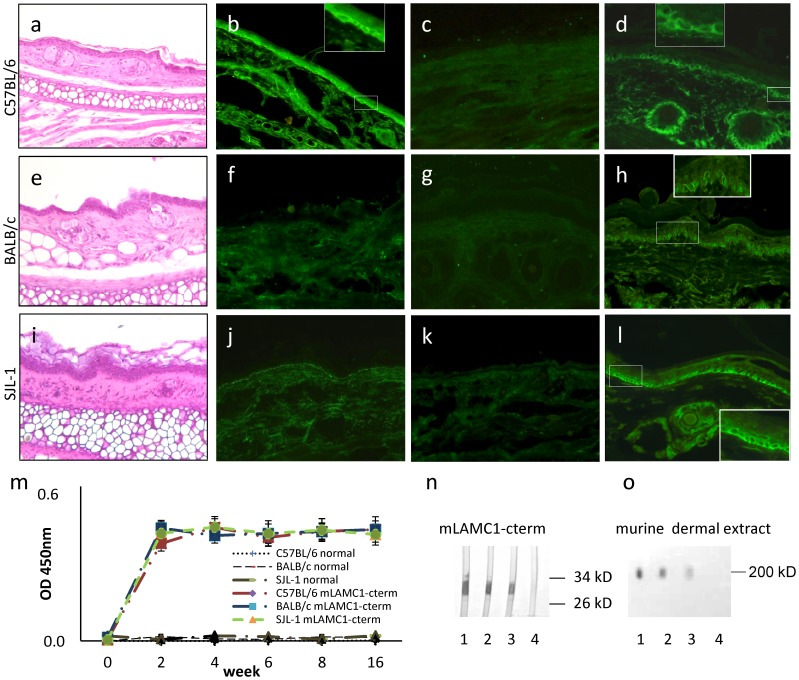
Immunization of mice with mLAMC1-cterm induces high serum levels of anti-mLAMC1-cterm antibodies but no skin lesions. Immunization of different mouse strains with the recombinant murine laminin γ1 C-terminus (mLAMC1-cterm) did induce high levels of anti-mLAMC1-cterm antibodies but no macro- or microscopic disease. Three different mouse strains (B57BL/6, BALB/c, and SJL; n = 5/strain) were immunized 4 times with 60 µg of recombinant mLAMC1-cterm in conjunction with TiterMax®. After 16 weeks, no clinical or histopathological changes (a, e, i) were seen. Deposition of mouse IgG at the dermal-epidermal junction (DEJ) was found in only 2 C56BL/6 mice (b). In all other mice, no IgG or C3 deposition was detected at the DEJ (f, j, c, g, k). In all mice, serum autoantibodies labeled the basal layer of keratinocytes at the murine DEJ (d, h, l) and reacted with recombinant mLAMC1-cterm by ELISA (m) and immunoblotting (n; C57BL/6, lane 1; BALB/c, lane 2; SJL, lane 3) and the 200 kDa p200 protein in extract of murine dermis (o; C57BL/6, lane 1; BALB/c, lane 2; SJL, lane 3). Serum of a mouse immunized with TiterMax® alone served as control (n and o; lane 4).

Following previous protocols [17], [18], [22], C57BL/6 (n = 5), BALB/c (n = 5), and SJL (n = 5) mice were immunized 4 times with mLAMC1-cterm. Control C57BL/6 (n = 1), BALB/c (n = 1), and SJL (n = 1) were immunized with an emulsion containing PBS and TiterMax®. After 16 weeks, no clinical or histopathological changes were detected in mLAMC1-cterm-immunized mice of the 3 strains (Fig. 7 a, e, i). By direct IF microscopy, 2 C57BL/6 showed faint deposits at the DEJ (Fig. 7 b). Murine C3 deposition at the DEJ was not observed (Fig. 7 c, g and k). In contrast, all mouse sera contained antibodies against recombinant mLAMC1-cterm (titre 1∶10^8^), the p200 protein in extract of murine dermis (titre 1∶5.000), and the DEJ (titer 1∶30) by ELISA, immunoblotting, and indirect IF microscopy, respectively (Fig. 7d, h, l-o).

## Discussion

In the present study, we showed that serum autoantibodies in anti-p200 pemphigoid are pathogenic while antibodies against laminin γ1 did not mediate this pathogenic effect in different experimental models.

In the first set of experiments, an ex vivo model of autoantibody-mediated leukocyte-dependent neutrophil activation and DES was applied [21]. As previously shown for IgG from patients with BP and EBA [10], [16], [23], all 7 anti-p200 pemphigoid patients sera induced DES. Recently, the C-terminus of laminin γ1 has been described as the immunodominant region in anti-p200 pemphigoid [6]. To dissect the impact of autoantibody reactivity against this autoantigenic site in our ex vivo model, IgG affinity-purified with the recombinant human C-terminus of laminin γ1 (hLAMC1-cterm) and serum depleted from hLAMC1-cterm-reactivity were prepared from anti-p200 pemphigoid sera. While latter serum fractions induced DES, hLAMC1-cterm-specific IgG was not pathogenic. The same results were obtained using IgG specific to the recombinant eukaryotic E8 fragment, a C-terminal portion of laminin 111 and specific to LAMC1-FL. When sera were completely depleted of IgG no DES occurred. This result clearly points to antibody-mediated tissue damage in anti-p200 pemphigoid.

The following hypotheses to explain inefficacy in our ex vivo model were compiled: i) the concentration of anti-LAMC1-cterm-IgG was too low, ii) glycosilation-dependent epitopes mediate pathogenicity, iii) the elution process of hLAMC1-cterm-specific IgG during the affinity purification procedure impaired pathogenicity, iv) antibodies against a heterotrimeric form of laminin γ1 are pathogenic, v) epitopes on laminin γ1 outside LAMC1-cterm are pathogenetically relevant, vi) the cryosection model is not suitable to demonstrate the pathogenic effect of anti-laminin γ1 antibodies, and vii) laminin γ1 is recognized by autoantibodies but not the autoantigen in anti-p200 pemphigoid.

To address the first hypothesis, a 5-fold higher IgG concentration compared to the corresponding patient sera as well as high concentrations of both rabbit IgG generated against murine LAMC1-cterm and commercially available anti-laminin γ1 IgG were used in the cryosection model, but no DES was observed. Since both the p200 protein and laminin γ1 were shown to be N-glycosylated [5], [6], we then investigated whether glycosilation-dependent epitopes mediate DES in our ex vivo model. When patient IgG, affinity purified against eukaryotic expressed hLAMC1-cterm was applied also no effect was observed. The third hypothesis claiming a potential damage of IgG following the elution process was excluded since hLAMC1-cterm-specific IgG showed strong reactivity with recombinant hLAMC1-cterm, the p200 protein in human dermis, and the human DEJ by immunoblotting and indirect IF microscopy, respectively. In addition, both rabbit serum and concentrated rabbit IgG generated against mLAMC1-cterm as well as a monoclonal anti-laminin γ1 antibody (all not subjected to affinity purification) were ineffective in the ex vivo model.

To test the fourth hypothesis we used the E8 fragment of laminin 111. This E8 fragment was used by Dainichi *et al.* for the detection of anti-laminin γ1 reactivity in anti-p200 pemphigoid sera [6]. Similar to experiments with hLAMC1-cterm E8 fragment-specific IgG was ineffective in the cryosection assay and serum depleted from anti-E8 antibodies was still reactive with the p200 protein in dermal extract and induced DES in our ex vivo model. To address the fifth hypothesis, epitope mapping experiments were performed that revealed the binding of only a minority of anti-p200 pemphigoid sera outside LAMC1-cterm. Thus, major pathogenic sites on laminin γ1 outside the LAMC1-cterm domain were excluded. Since the recombinant fragments used in the epitope mapping studies were overlapping by only 0 to 5 amino acids and some B-cell epitopes could thus have been missed patients’ IgG affinity-purified with the entire laminin γ1 chain was employed in the cryosection model. Again, the anti-full length laminin γ1 IgG did not induce DES while IgG depleted from anti-full length laminin γ1 retained its DES-inducing capacity.

In a final set of experiments to address the sixth hypothesis, the effect of anti-LAMC1-cterm antibodies was explored in various *in vivo* models. Since patient antibodies do not cross-react with murine skin [19], antibodies against murine LAMC1-cterm were raised by immunization of rabbits and different mouse strains. High doses of total IgG from these rabbits and IgG affinity-purified using recombinant mLAMC1-cterm were then injected into adult and neonatal mice, respectively. While high levels of circulating anti-mLAMC1-cterm antibodies were present in all animals, binding of IgG at the DEJ was weak, did not attract C3 at the DEJ, and did not result in clinical or histopathological lesions. Similar findings were obtained when mice from different strains were immunized with mLAMC1-cterm. In contrast, previously, injection of rabbit IgG raised against the immunodominant regions of type XVII collagen (BP180) into neonatal mice reproduced the human disease (BP) within 48 hours [9]. Also, injection of rabbit or human IgG against the immunodominant regions of type XVII collagen or type VII collagen into adult mice recapitulated the human diseases BP and EBA, respectively [11], [13], [24]. Recently, we successfully induced clinical disease mimicking the human disorders BP and EBA by immunizing susceptible mice strains with the immunodominant regions of type XVII collagen and type VII collagen, respectively, following a similar protocol as applied in the present study [11], [18]. Autoimmunity against laminin 332, the target antigen in a subgroup of patients with mucous membrane pemphigoid was induced in mice by the passive transfer of human and rabbit anti-laminin 332 IgG [14], [25]. These data show that autoimmunity against target antigens including collagens and laminin could be mounted by both passive transfer of autoantibodies and immunization-induced autoantibodies mimicking the clinical disease of the corresponding subepidermal blistering diseases. We speculate that the failure to induce clinical disease in our mouse models, although high levels of serum anti-mLAMC1-cterm IgG were present, may be attributed to the weak binding of autoantibodies at the DEJ. This notion is fuelled by the peculiar binding pattern of anti-LAMC1-cterm IgG at the basal keratinocytes of the DEJ that differs from the exclusively linear binding observed in patients’ skin and of patients’ serum autoantibodies labeling human skin. Furthermore, the unique reactivity of patient autoantibodies with the DEJ, but not with any other tissues which express the laminin γ1 chain, remains enigmatic.

Based on these data, we conclude that while the C-terminus of laminin γ1 is a major target of autoantibodies in anti-p200 pemphigoid, we failed to demonstrate the pathogenicity of anti-laminin γ1 antibodies using different approaches. A somehow similar autoantibody constellation is known in BP. While BP230 had been described as the major target antigen recognized by the majority of BP patients [26], [27], its pathogenic relevance could not be unequivocally demonstrated. Subsequently, antibodies against type XVII collagen were described [28], [29] and numerous evidence for their pathogenic relevance has been gathered [9], [10], [20], [24], [30], [31].

In summary, in this study, the pathogenic potential of autoantibodies in anti-p200 pemphigoid was shown for the first time. The specificity of the pathogenically relevant autoantibody awaits further elucidation.

## References

[1] Zillikens D, Kawahara Y, Ishiko A, Shimizu H, Mayer J (1996). A novel subepidermal blistering disease with autoantibodies to a 200-kDa antigen of the basement membrane zone.. J Invest Dermatol.

[2] Chen KR, Shimizu S, Miyakawa S, Ishiko A, Shimizu H (1996). Coexistence of psoriasis and an unusual IgG-mediated subepidermal bullous dermatosis: identification of a novel 200-kDa lower lamina lucida target antigen.. Br J Dermatol.

[3] Dilling A, Rose C, Hashimoto T, Zillikens D, Shimanovich I (2007). Anti-p200 pemphigoid: a novel autoimmune subepidermal blistering disease.. J Dermatol.

[4] Zillikens D, Ishiko A, Jonkman MF, Chimanovitch I, Shimizu H (2000). Autoantibodies in anti-p200 pemphigoid stain skin lacking laminin 5 and type VII collagen.. Br J Dermatol.

[5] Shimanovich I, Hirako Y, Sitaru C, Hashimoto T, Brocker EB (2003). The autoantigen of anti-p200 pemphigoid is an acidic noncollagenous N-linked glycoprotein of the cutaneous basement membrane.. J Invest Dermatol.

[6] Dainichi T, Kurono S, Ohyama B, Ishii N, Sanzen N (2009). Anti-laminin gamma-1 pemphigoid.. Proc Natl Acad Sci U S A.

[7] Dainichi T, Koga H, Tsuji T, Ishii N, Ohyama B (2010). From anti-p200 pemphigoid to anti-laminin gamma1 pemphigoid.. J Dermatol.

[8] Groth S, Recke A, Vafia K, Ludwig RJ, Hashimoto T (2011). Development of a simple enzyme-linked immunosorbent assay for the detection of autoantibodies in anti-p200 pemphigoid.. Br J Dermatol.

[9] Liu Z, Diaz LA, Troy JL, Taylor AF, Emery DJ (1993). A passive transfer model of the organ-specific autoimmune disease, bullous pemphigoid, using antibodies generated against the hemidesmosomal antigen, BP180.. J Clin Invest.

[10] Sitaru C, Schmidt E, Petermann S, Munteanu LS, Brocker EB (2002). Autoantibodies to bullous pemphigoid antigen 180 induce dermal-epidermal separation in cryosections of human skin.. J Invest Dermatol.

[11] Sitaru C, Mihai S, Otto C, Chiriac MT, Hausser I (2005). Induction of dermal-epidermal separation in mice by passive transfer of antibodies specific to type VII collagen.. J Clin Invest.

[12] Woodley DT, Chang C, Saadat P, Ram R, Liu Z (2005). Evidence that anti-type VII collagen antibodies are pathogenic and responsible for the clinical, histological, and immunological features of epidermolysis bullosa acquisita.. J Invest Dermatol.

[13] Woodley DT, Ram R, Doostan A, Bandyopadhyay P, Huang Y (2006). Induction of epidermolysis bullosa acquisita in mice by passive transfer of autoantibodies from patients.. J Invest Dermatol.

[14] Lazarova Z, Yee C, Darling T, Briggaman RA, Yancey KB (1996). Passive transfer of anti-laminin 5 antibodies induces subepidermal blisters in neonatal mice.. J Clin Invest.

[15] Yamamoto K, Inoue N, Masuda R, Fujimori A, Saito T (2002). Cloning of hamster type XVII collagen cDNA, and pathogenesis of anti-type XVII collagen antibody and complement in hamster bullous pemphigoid.. J Invest Dermatol.

[16] Sitaru C, Kromminga A, Hashimoto T, Brocker EB, Zillikens D (2002). Autoantibodies to type VII collagen mediate Fcgamma-dependent neutrophil activation and induce dermal-epidermal separation in cryosections of human skin.. Am J Pathol.

[17] Sitaru C, Chiriac MT, Mihai S, Buning J, Gebert A (2006). Induction of complement-fixing autoantibodies against type VII collagen results in subepidermal blistering in mice.. J Immunol.

[18] Hirose M, Recke A, Beckmann T, Shimizu A, Ishiko A (2011). Repetitive immunization breaks tolerance to type XVII collagen and leads to bullous pemphigoid in mice.. J Immunol.

[19] Hofmann SC, Voith U, Sasaki T, Trueb RM, Nischt R (2008). The autoantigen in anti-p200 pemphigoid is synthesized by keratinocytes and fibroblasts and is distinct from nidogen-2.. J Invest Dermatol.

[20] Schmidt E, Reimer S, Kruse N, Jainta S, Brocker EB (2000). Autoantibodies to BP180 associated with bullous pemphigoid release interleukin-6 and interleukin-8 from cultured human keratinocytes.. J Invest Dermatol.

[21] Gammon WR, Merritt CC, Lewis DM, Sams WM, Carlo JR (1982). An in vitro model of immune complex-mediated basement membrane zone separation caused by pemphigoid antibodies, leukocytes, and complement.. J Invest Dermatol.

[22] Ludwig RJ, Recke A, Bieber K, Muller S, Marques Ade C (2011). Generation of antibodies of distinct subclasses and specificity is linked to H2s in an active mouse model of epidermolysis bullosa acquisita.. J Invest Dermatol.

[23] Mihai S, Chiriac MT, Herrero-Gonzalez JE, Goodall M, Jefferis R (2007). IgG4 autoantibodies induce dermal-epidermal separation.. J Cell Mol Med.

[24] Nishie W, Sawamura D, Goto M, Ito K, Shibaki A (2007). Humanization of autoantigen.. Nat Med.

[25] Lazarova Z, Hsu R, Yee C, Yancey KB (2000). Human anti-laminin 5 autoantibodies induce subepidermal blisters in an experimental human skin graft model.. J Invest Dermatol.

[26] Mueller S, Klaus-Kovtun V, Stanley JR (1989). A 230-kD basic protein is the major bullous pemphigoid antigen.. J Invest Dermatol.

[27] Stanley JR, Hawley-Nelson P, Yuspa SH, Shevach EM, Katz SI (1981). Characterization of bullous pemphigoid antigen: a unique basement membrane protein of stratified squamous epithelia.. Cell.

[28] Diaz LA, Ratrie H, 3rd, Saunders WS, Futamura S, Squiquera HL, *et al* (1990). Isolation of a human epidermal cDNA corresponding to the 180-kD autoantigen recognized by bullous pemphigoid and herpes gestationis sera. Immunolocalization of this protein to the hemidesmosome.. J Clin Invest.

[29] Labib RS, Anhalt GJ, Patel HP, Mutasim DF, Diaz LA (1986). Molecular heterogeneity of the bullous pemphigoid antigens as detected by immunoblotting.. J Immunol.

[30] Schmidt E, Obe K, Brocker EB, Zillikens D (2000). Serum levels of autoantibodies to BP180 correlate with disease activity in patients with bullous pemphigoid.. Arch Dermatol.

[31] Iwata H, Kamio N, Aoyama Y, Yamamoto Y, Hirako Y (2009). IgG from patients with bullous pemphigoid depletes cultured keratinocytes of the 180-kDa bullous pemphigoid antigen (type XVII collagen) and weakens cell attachment.. J Invest Dermatol.

